# Implementation Study of Patient-Ready Syringes Containing 25 mg/mL Methotrexate Solution for Use in Treating Ectopic Pregnancy

**DOI:** 10.1155/2014/689308

**Published:** 2014-05-12

**Authors:** R. Respaud, A. S. Gaudy, C. Arlicot, J. F. Tournamille, M. C. Viaud-Massuard, C. Elfakir, D. Antier

**Affiliations:** ^1^EA 6306, IMT, Laboratoire de Chimie Organique, Faculté de Pharmacie, Université de Tours, 31 Avenue Monge, 37200 Tours, France; ^2^Pharmacie, CHRU de Tours, 2 Boulevard Tonnellé, 37044 Tours Cedex 9, France; ^3^Pôle de Gynécologie Obstétrique, Médecine Foetale Médecine et Biologie de la Reproduction, Centre Olympe-de-Gouges, CHRU de Tours, 2, Boulevard Tonnellé, 37044 Tours Cedex 9, France; ^4^Institut de Chimie Organique et Analytique (ICOA) UMR CNRS 7311, Université d'Orléans, BP 6759, 45067 Orléans Cedex 2, France

## Abstract

*Background.* Ectopic pregnancy (EP) is a significant cause of morbidity and mortality during the first trimester of pregnancy. Small unruptured tubal pregnancies can be treated medically with a single dose of methotrexate (MTX). *Objective.* The aim of this study was to evaluate the stability of a 25 mg/mL solution of MTX to devise a secure delivery circuit for the preparation and use of this medication in the management of EP. *Method.* MTX solutions were packaged in polypropylene syringes, stored over an 84-day period, and protected from light either at +2 to +8°C or at 23°C. We assessed the physical and chemical stability of the solutions at various time points over the storage period. A pharmaceutical delivery circuit was implemented that involved the batch preparation of MTX syringes. *Results.* We show that 25 mg/mL MTX solutions remain stable over an 84-day period under the storage conditions tested. Standard doses were prepared, ranging from 50 mg to 100 mg. The results of this study suggest that MTX syringes can be prepared in advance by the pharmacy, ready to be dispensed at any time that a diagnosis of EP is made. *Conclusion.* The high stability of a 25 mg/mL MTX solution in polypropylene syringes makes it possible to implement a flexible and cost-effective delivery circuit for ready-to-use preparations of this drug, providing 24-hour access and preventing treatment delays.

## 1. Introduction

Ectopic pregnancy (EP) is a potentially life threatening complication of early pregnancy. In developed countries, 1.3–2% of all pregnancies are extrauterine [1]. Several risk factors have been identified including pelvic inflammatory disease (especially due to chronic* Chlamydia* infection), tubal abnormalities, smoking, the use of an intrauterine device, and a history of EP [2, 3].

The most serious complication of EP is tubal rupture, which can lead to catastrophic haemorrhage and shock [2, 3]. Once an EP is diagnosed, it can be treated in three ways: surgical treatment, medical treatment with methotrexate injection, or expectant management.

For small, stable EPs, there is no difference in subsequent fertility between patients treated with methotrexate and those treated by surgery [2]; therefore, if serum hCG concentrations are low (under 5.000 UI/L) and if the patient shows no major bleeding in the peritoneal cavity and agrees to close follow-up medical treatment with methotrexate should be the preferred method. Methotrexate is a folic acid antagonist that binds to dihydrofolate reductase, and it has been used as a first-line therapy for suitable unruptured EPs since 1982 [4]. Methotrexate is safe and almost no adverse effects have been reported on the reproductive outcome [5]. The overall success rate of medical treatment in appropriately selected women is nearly 90% [1]. The most accepted approach to therapy is to administer a single low intramuscular dose of methotrexate: 1 mg/kg of body weight or 50 mg/m² (without folinic acid). A single-dose regimen was introduced to minimize side effects, to improve patient compliance, and to reduce overall costs [5]. For the treatment of EPs, methotrexate is administered by a syringe containing a 25 mg/mL solution, but appropriate prefilled syringes are not commercially available.

In most hospitals, the reconstitution and preparation of anticancer drugs, such as methotrexate, now take place in centralized compounding units in a controlled environment with appropriately trained expert staff [6]. Several aspects must be taken into account when such drugs are prepared: dose accuracy, sterility, occupational exposure, and drug stability. This ensures that only products that are safe from a bacteriological, dosage accuracy, and contamination point of view are dispensed. Thus, these patient-ready preparations are prepared under Good Hospital Pharmacy Manufacturing Practice (GHPMP) conditions, which involves the principles of GMP applied to Hospital Pharmacy Compounding [6]. The only relevant issue is therefore the actual chemical and physical stability of the drug formulation. At Tours University Hospital, we have opted for a system of dose banding from batch preparations involving syringes of 25 mg/mL methotrexate, to ensure the safety of both patients and nursing staff during the treatment of EP. The solution is available in 20 mL vials, but administering this formulation involves a nurse handling a cytotoxic and potentially teratogenic solution and exposes the patient to potential dosing error [7].

No data is available about the stability of 25 mg/mL MTX in polypropylene syringes. Therefore, we decided to examine the physicochemical stability of the preparation and design a protocol for the preparation and delivery of MTX intended for the treatment of EPs in the gynaecology department.

## 2. Methods

### 2.1. Stability Study

#### 2.1.1. Material

The 25 mg/mL methotrexate and polypropylene syringes were provided by our local pharmacy.

#### 2.1.2. Preparation of the Syringes

Solutions of methotrexate (25 mg/mL) were packaged in polypropylene syringes without further dilution. All stages of the preparation were conducted under GHPMP conditions. Triplicate samples were prepared for each evaluation time point and were stored for 84 days at +2 to +8°C protected from light and for 84 days at 23°C protected from light with overwraps from Bexen. Physical and chemical stability studies were performed at the time of sample preparation and after 1, 7, 14, 28, 56, and 84 days of storage.

#### 2.1.3. Physical and Chemical Stability

The physical stability of the methotrexate solution was assessed by determining its turbidity. The turbidity of each sample was measured with a spectrophotometer at two different wavelengths (550 and 790 nm). Triplicate analyses were performed on each sample at each wavelength. Physical instability was defined as a change in turbidity ≥1.96 CV%.

The chemical stability of methotrexate solution was assessed by measuring its concentration with an HPLC-UV-ELSD method. The HPLC analytical method used was adapted from a method developed for pemetrexed and its accuracy, precision, specificity, and linearity were validated for use in this study according to ICH guidelines [8]. All samples were diluted prior to analysis with water to a nominal methotrexate concentration of 0.1 mg/mL. HPLC was performed twice for each of the triplicate samples. The initial concentration of methotrexate in the samples was defined as 100%, and subsequent sample concentrations were expressed as a percentage of this initial concentration. The methotrexate was defined as stable if no less than 95% of the initial drug concentration remained in the solution at the end of the 84-day period.

#### 2.1.4. Pharmaceutical Dispensing Circuit

*Dose Banding*. The syringes were prepared by expert staff in a centralized compounding unit in a controlled and validated environment. The principles of GHPMP are applied in this centralized unit, which ensures sterile conditions during the preparation process and therefore the sterility of the syringes produced. The predetermined standard doses of methotrexate were determined in agreement with care unit physicians of the Teaching Hospital of Tours. The following range of standard doses, based on 1 mg/kg body weight as recommended by the “Collège National des Gynécologues Obstétriciens Francais,” was selected for preparation: 50, 60, 70, 80, 90, and 100 mg. Patients weighing less than 50 kg receive a dose of 50 mg, because the low toxicity of methotrexate at these doses permits an approximation of the dosage used [3, 5]. In obese women, the maximum dose is capped at 100 mg [9]. The same range of standard doses can be used based on 50 mg/m² posology of MTX.

## 3. Results

### 3.1. Stability Study

The 25 mg/mL methotrexate solutions packaged in polypropylene syringes were clear both at room temperature and following refrigeration. We observed no change in the turbidity measurements (≥1.96 CV%) and no change in pH measurements (≥1.96 CV%) during the study (84 days) (data not shown). Each sample was analysed by HPLC after suitable dilution in ultrapure water as required for the concentration assay. The percentages of the initial concentration of methotrexate found in the solutions after various periods of storage in polypropylene syringes and protected from light are listed in Table 1. We observed no significant disappearance of the drug (exceeding 5%) in the syringes over an 84-day period. Furthermore, we detected no major degradation products by UV or ELSD during the study.

### 3.2. Pharmaceutical Dispensing Circuit

**Figure 1:**
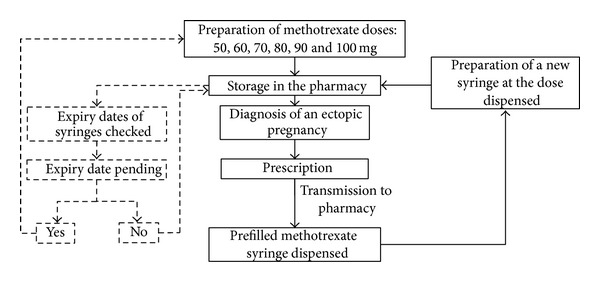
Pharmaceutical dispensing circuit.

Our findings have made it possible to introduce a dispensing circuit for pharmacy-prepared dose-banding syringes of methotrexate solution (Figure 1). All syringes are prepared in advance, during the usual opening hours of the unit. Methotrexate expiry dates are checked once a month. All dose-banded syringe batches approaching their expiry date are identified and replaced.

## 4. Discussion

The medical management of EP with methotrexate is a highly successful treatment strategy for suitably selected EPs. Indeed, it is a cost-effective alternative to laparoscopic surgery when serum hCG concentrations are low (under 5.000 UI/L), without major bleeding in the peritoneal cavity, and when the patient complies with close follow-up [2]. Methotrexate is administered by a polypropylene syringe containing a 25 mg/mL solution, but such syringes are not commercially available. Prefilled syringes of methotrexate solution are indeed marketed (Metoject in France) at a concentration of 10 mg/mL or 50 mg/mL for rheumatoid arthritis treatment, but with these syringes, the treatment of EP would require between one and four injections. With syringes containing 25 mg/2.5 mL, the total volume to be injected (4–10 mL in most cases) exceeds the maximum volume that can be administered intramuscularly at a single injection site (5 mL) [10]. Methotrexate 25 mg/mL solution is also available in 20 mL vials, but prior to administration, it has to be handled by the nursing staff, who may be inexperienced in handling cytotoxic solutions.

Moreover, obliging nurses to administer multiple injections, to calculate the volume to be injected, and to handle methotrexate solutions is a possible source of error. At Tours University Hospital, prefilled syringes containing methotrexate for use in EPs are prepared by the pharmacy to avoid the risk of dosing errors and handling exposures. This ensures the safety of both the nursing staff and patients, because the pharmacy staff are well trained in preparing cytotoxic medications, and the final preparation is sterile, is controlled (drug identification, dose, and volume), and has a concentration suitable for the treatment of ectopic pregnancies [6]. However, the pharmacy unit is only open during the day, so patients with an ectopic pregnancy presenting outside these hours have to return later, which is both physically inconvenient and psychologically distressing, especially for a potentially life-threatening condition. Another possibility would be to mobilise the on-call team, but this would increase both patient waiting time and the cost of the preparations. Furthermore, the unit cost of preparing a series of methotrexate doses is lower than that of preparing single doses in an emergency. These factors explain why preparing ready-to-use syringes to ensure 24/7 availability of MTX is preferable for everyone: patients, the care unit, and the pharmacy unit.

We found that MTX solutions are stable in the described storage conditions; thus, standard doses of methotrexate may be prepared in advance. Preparation of a range of patient-ready standard doses of methotrexate allows the pharmacy to supply appropriate and safe treatment without delay when an ectopic pregnancy is diagnosed. This dispensing circuit also improves pharmacy capacity planning.

## 5. Conclusion

The Department of Pharmacy of the Tours University Hospital has implemented a protocol for the preparation of ready-to-use methotrexate. This combines simplicity and safety for both patients and the clinical ward, and it is also a simple and cost-saving measure for the hospital. The demonstrated stability of this 25 mg/mL methotrexate solution over a period of 84 days guarantees cost-effective and safe patient care.

## Figures and Tables

**Table 1:** Stability of 25 mg/mL methotrexate solution for injection packaged in polypropylene syringes.

Time (days)	% of initial concentration remaining (mean ± SD; CV%) (*n* = 3)	
	25 mg/mL	25 mg/mL
	23°C dark	+2 to +8°C dark
1	101.2 ± 1.6; 1.6	101.2 ± 0.3; 0.3
7	101.2 ± 0.3; 0.3	100.3 ± 1.0; 1.0
14	100.0 ± 1.0; 1.0	99.0 ± 2.3; 2.3
28	100.0 ± 0.8; 0.8	99.2 ± 0.3; 0.3
56	100.3 ± 0.8; 0.8	99.0 ± 0.5; 0.5
84	102.2 ± 1.1; 1.0	102.5 ± 2.3; 2.2
